# Highly pathogenic avian influenza A(H5N1) virus of clade 2.3.4.4b isolated from a human case in Chile causes fatal disease and transmits between co-housed ferrets

**DOI:** 10.1080/22221751.2024.2332667

**Published:** 2024-03-17

**Authors:** Joanna A. Pulit-Penaloza, Nicole Brock, Jessica A. Belser, Xiangjie Sun, Claudia Pappas, Troy J. Kieran, Poulami Basu Thakur, Hui Zeng, Dan Cui, Julia Frederick, Rodrigo Fasce, Terrence M. Tumpey, Taronna R. Maines

**Affiliations:** a Influenza Division, NCIRD, Centers for Disease Control and Prevention, Atlanta, GA, USA; b Viral Diseases Sub Department, Public Health Institute, ISP, Santiago, Chile

**Keywords:** Ferret, avian influenza, transmission, A(H5N1), clade 2,3,4,4b

## Abstract

Clade 2.3.4.4b highly pathogenic avian influenza A(H5N1) viruses have caused large outbreaks within avian populations on five continents, with concurrent spillover into a variety of mammalian species. Mutations associated with mammalian adaptation have been sporadically identified in avian isolates, and more frequently among mammalian isolates following infection. Reports of human infection with A(H5N1) viruses following contact with infected wildlife have been reported on multiple continents, highlighting the need for pandemic risk assessment of these viruses. In this study, the pathogenicity and transmissibility of A/Chile/25945/2023 HPAI A(H5N1) virus, a novel reassortant with four gene segments (PB1, PB2, NP, MP) from North American lineage, isolated from a severe human case in Chile, was evaluated in vitro and using the ferret model. This virus possessed a high capacity to cause fatal disease, characterized by high morbidity and extrapulmonary spread in virus-inoculated ferrets. The virus was capable of transmission to naïve contacts in a direct contact setting, with contact animals similarly exhibiting severe disease, but did not exhibit productive transmission in respiratory droplet or fomite transmission models. Our results indicate that the virus would need to acquire an airborne transmissible phenotype in mammals to potentially cause a pandemic. Nonetheless, this work warrants continuous monitoring of mammalian adaptations in avian viruses, especially in strains isolated from humans, to aid pandemic preparedness efforts.

## Introduction

Highly pathogenic avian influenza (HPAI) viruses of clade 2.3.4.4 first gained attention in 2014 due to their extensive capacity for reassortment and spread, giving rise to A(H5Nx) viruses, notably A(H5N2), A(H5N5), A(H5N6), and A(H5N8), bearing a variety of gene constellations and causing large outbreaks on multiple continents [1]. While A(H5Nx) outbreaks to date have been mainly restricted to wild and domestic birds, clade 2.3.4.4b A(H5N1) viruses have recently displayed an increased capability to infect mammalian species. The widespread detection of A(H5N1) viruses in birds across five continents, and spillover to mammalian species (including humans) represent a global public health concern [2]. Reports of outbreaks in farmed mink in Spain [3], New England harbour and grey seals in the United States of America [4], sea lions in Peru and Chile [5], and cats in France and Poland [6–8] suggest that clade 2.3.4.4b A(H5N1) viruses could be evolving towards more efficient mammalian transmission, a capacity previously not associated with A(H5N1) viruses. This has been supported by reports of human cases following exposure to contaminated environments or animals infected with clade 2.3.4.4b A(H5N1) viruses [9]*.* While frequent human infections and sustained human-to-human transmission have not been reported, the increased adaptability of clade 2.3.4.4b avian H5 viruses to mammalian species, as well as the detection of mammalian adaptation markers in the sequences of many of the viruses isolated from mammals, necessitates heightened surveillance systems to closely monitor the genetic and pathobiological characteristics of these influenza viruses in order to detect possible changes that could lead to efficient replication in the human respiratory tract and onward transmission leading to a pandemic.

The use of the ferret model has been instrumental in assessing the relative pathogenicity and transmissibility of HPAI A(H5Nx) viruses circulating in avian populations over the last decade. Reassortant clade 2.3.4.4 A(H5N2) and A(H5N8) viruses from the 2014–2015 outbreaks displayed generally mild disease in mammalian models and lacked the ability to transmit between ferrets [10–13]. However, some clade 2.3.4.4 A(H5N6) viruses that crossed the species barrier and infected humans had the ability to cause severe disease in ferrets and exhibited limited transmissibility between co-housed ferrets [14, 15]. Early 2.3.4.4b virus isolates detected in North America at the end of 2021 also displayed a low pathogenicity phenotype in ferrets. However, following reassortment with viruses circulating in wild birds in North America and the acquisition of different combinations of ribonucleoprotein and NP genes, a virus capable of increased pathogenicity and neurologic involvement in ferrets was recently identified; this virus did not possess the ability to transmit between ferrets [16, 17]. These studies highlight the heterogeneity of currently circulating viruses and support the need for in vivo assessments of novel reassortant viruses, including human isolates, for pandemic risk assessment purposes.

Between January 2022 and September 2023, there have been at least 12 reported human infections with clade 2.3.4.4b A(H5N1) viruses [9]. HPAI A(H5N1) A/Chile/25945/2023 (Chile/25945) virus was isolated in Chile from a 53-year-old man with no comorbidities. The patient initially presented mild signs of infection including cough and sore throat, followed by admission to the hospital due to severe pneumonia necessitating mechanical ventilation [18]. Analysis of the Chile/25945 A(H5N1) virus sequence revealed that this virus acquired four genes (PB2, PB1, NP, MP) of North American lineages and possessed several mammalian adaptation markers [19]. Two amino acid substitutions found in PB2, Q591 K and D701N, have been previously associated with increased polymerase activity in mammalian cell lines and increased virulence in mammalian models [20]. These observations suggested that the reassortant Chile/25945 H5N1 virus may have an increased ability to replicate in mammalian cells and have an increased pathogenicity in the mammalian animal model. While assessments of viruses isolated from avian and mammalian species infected by clade 2.3.4.4b A(H5N1) viruses have been conducted, human isolates have yet to be similarly studied. To address this gap in knowledge, we evaluated the replication efficacy of the Chile/25945 virus in human bronchial epithelial cells (Calu-3) and assessed the pathogenesis and transmissibility of this virus using the ferret model. We found that the viruses replicated very efficiently in mammalian cells and possessed a high capacity to cause fatal disease in the ferret model. Importantly, the virus was transmitted to naïve contacts in a direct contact setting, with contact animals exhibiting severe and fatal disease.

## Results

### HPAI A(H5N1) virus replication in a human respiratory tract cell line

Clade 2.3.4.4 HPAI A(H5N1) viruses have demonstrated a capacity to replicate to high titre in multiple in vitro systems emulative of and/or derived from the human respiratory tract [10, 16], but a side-by-side comparison of relative replication capacity between human and zoonotic 2.3.4.4b isolates has not been conducted. We assessed the ability of Chile/25945 virus to replicate in Calu-3 cells grown under air-liquid interface conditions as compared with a panel of four zoonotic A(H5N1) viruses, inclusive of both avian (A/bald eagle/Florida/22-006544-004/2022) and wild mammalian (A/harbour seal/Maine/22-020983-002/2022, A/Virginia opossum/Iowa/22-016780-001/2022, and A/fox/Wisconsin/22-013774-002/2022) isolates representative of multiple genotypes of recently detected viruses in North America.

**Figure 1.**
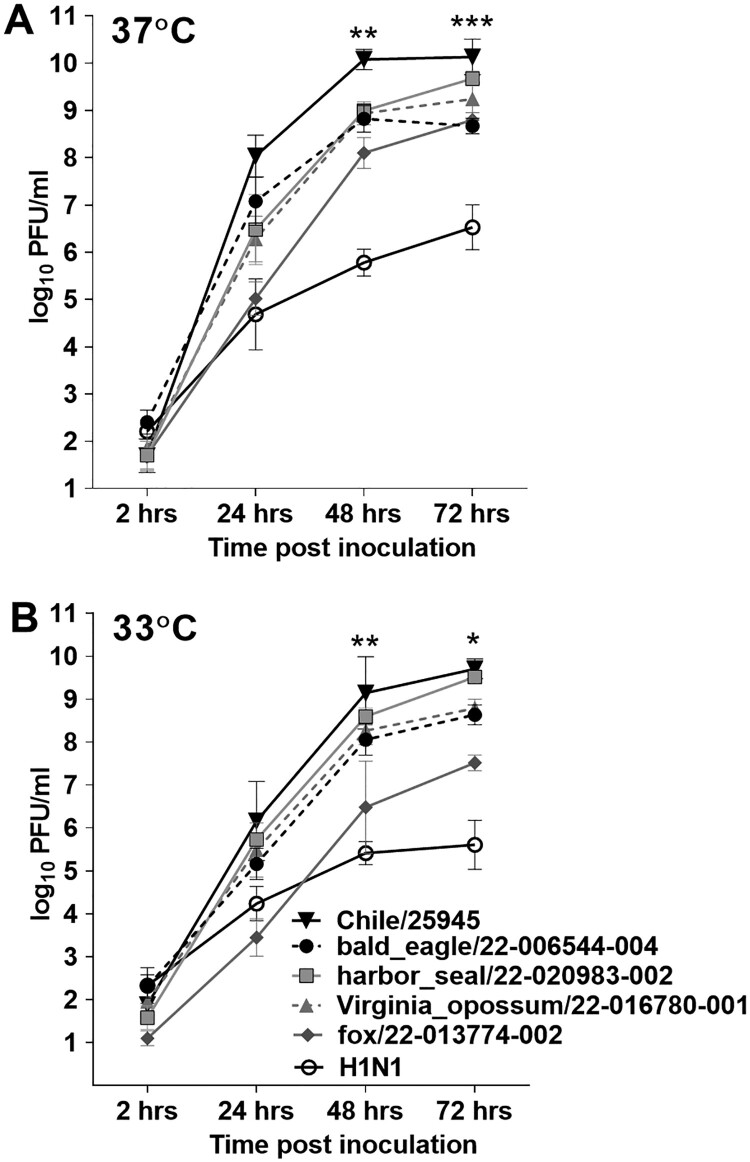
**Replication kinetics of clade 2.3.4.4b viruses in Calu-3 cells.** Replication kinetics of A(H5N1) viruses A/bald eagle/Florida/22-006544-004/2022, A/harbour seal/Maine/22-020983-002/2022, A/Virginia opossum/Iowa/22-016780-001/2022, A/fox/Wisconsin/22-013774-002/2022, and A/Chile/25945/2023 were evaluated in human respiratory tract cells and compared with the A(H1N1)pdm09 strain A/Michigan/45/2015 (MI/45). Calu-3 cells were grown to confluence under air-liquid interface conditions in 12-mm–diameter transwell inserts. The cells were infected apically at a multiplicity of infection of 0.01 for 1 hr, and then washed with media and incubated at 37°C **(A)** or 33°C **(B).** Samples were collected at indicated times post-infection and titrated in MDCK cells by a standard plaque assay. The limit of virus detection was 1 log_10_ PFU/ml. Error bars indicate standard deviation. Statistical significance between the titres of the Chile/25945 virus compared to other viruses at each time point was analyzed by two-way analysis of variance (ANOVA) with Tukey’s post-test; * *p* < 0.05, ** *p* < 0.01, *** *p* < 0.001.

A(H5N1) viruses isolated from avian, human, or zoonotic mammalian species were all capable of high-titre replication in Calu-3 cells, reaching peak mean titres >8.5 log_10_ PFU or >7.5 log_10_ PFU/ml when cultured at 37°C or 33°C, respectively. Peak mean titres for all clade 2.3.4.4b viruses were higher than those reached by a representative A(H1N1)pdm09 human isolate (A/Michigan/15/2015) at either culture temperature, further supporting the replicative ability of A(H5N1) viruses isolated from diverse hosts to reach high viral titres in cells emulative of the human airways. However, the human isolate Chile/25945 nonetheless reached higher titres early after infection, with the difference in titres reaching statistical significance compared with all other A(H5N1) viruses tested at 48 and 72 hrs p.i. at 37°C (p 0.0016-<0.0001 by two-way ANOVA with Tukey’s pos-test). Generally comparable peak viral titres among all A(H5N1) viruses at temperatures emulative of the human upper (33°C) and lower (37°C) airways, and the enhanced capacity for Chile/25945 virus to replicate efficiently early after infection relative to other zoonotic isolates, support the capacity of clade 2.3.4.4b viruses to replicate efficiently in the mammalian upper respiratory tract, prompting subsequent in vivo evaluation of Chile/25945 virus (Figure 1).

### Pathogenesis of HPAI A(H5N1) human isolate in ferrets

**Figure 2.**
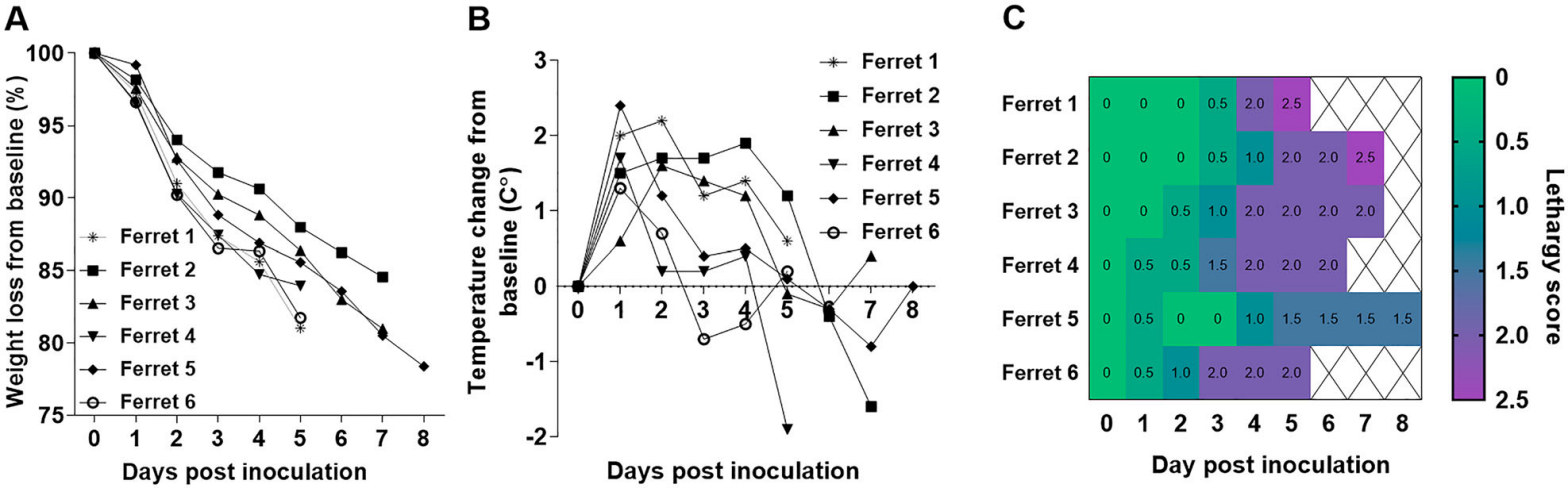
**Changes in body weight, temperature, and lethargy in ferrets inoculated with HPAI A/Chile/25945/2023 A(H5N1) virus.** Percent weight loss from pre-inoculation baseline body weight (A). Body temperature change from pre-inoculation baseline temperature (B). Lethargy was evaluated based on a scoring system of 0–3, which was used to calculate a relative inactivity index (C). Time courses for individual ferrets are shown up to the day of euthanasia (days 5-8).

To assess the capacity of the Chile/25945 A(H5N1) virus to cause severe disease in ferrets, animals were inoculated with 6 log_10_ EID_50_ of virus and observed daily for clinical signs of infection. Ferrets displayed increased body temperature (mean max 1.9°C over baseline) and rapid weight loss post-inoculation (p.i.) (Figure 2 A and B). In addition, other signs of disease, including nasal discharge, diarrhea, and ocular discharge, were observed. Increasing lethargy and severe and sustained clinical signs of infection, including laboured breathing, necessitated humane euthanasia of all the inoculated animals between days 5–8 p.i. (Figure 2 C and Table 1). Serially collected nasal washes (NW) revealed the capacity for high titre replication of virus throughout the acute period of infection. NW titres peaked between days 2–3 p.i. with a mean titre of 7.5 log_10_ EID_50_/ml and lasted through day 7 p.i. Detection of infectious virus in rectal swab (RS) specimens was observed in samples from 4/6 of the inoculated animals at a maximum mean titre of 2.9 log_10_ EID_50_/ml, and from a conjunctival wash (CW) specimen from one of the six inoculated animals (2.5 log_10_ EID_50_/ml), supporting the capacity of this virus to spread beyond the respiratory tract.

**Table 1.** Summary results of pathogenesis and transmission of A/Chile/25945/2023 A(H5N1) virus in ferrets.

Ferret group ^a^	NW titre ^b^	RS titre ^b^	Weight loss (%) ^c^	Temp increase (C°) ^d^	Leth ^e^	Nasal disch. ^f^	Ocular disch ^f^	Diarrh.^f^	Mortality^g^	Sero conv.^h^
Inoculated	7.5 ± 1.1; 6/6	2.9 ± 0.4 (4/6)	18.2 (6/6)	1.9 (6/6)	2.2	5/6	2/6	6/6	6/6 (5-8)	NT*
DC	5.4 ± 2.2; 2/3	NT	15.1 (2/3)	1.8 (2/3)	1.7	1/3	0/3	1/3	2/3 (9,10)	1/1*
RDC	0/3	NT	0/3	0/3	0/3	0/3	0/3	0/3	0/3	0/3
FC	0/3	NT	0/3	0/3	0/3	0/3	0/3	0/3	0/3	0/3

^a^ Ferret groups: inoculated – ferrets intranasally inoculated with 6 log_10_ EID_50_/ml; DC- direct contact ferrets; RDC-respiratory droplet contact ferrets; FC-fomite contact ferrets.

^b^ Average maximum nasal wash (NW) and rectal swab (RS) titres expressed as log_10_ EID_50_/ml- ± SD among ferrets with detectable titre. The number of ferrets with detectable virus over the total number of ferrets is in parenthesis.

^c^ Average weight loss on the day of euthanasia. Number of ferrets that displayed weight loss over the total number of animals is in parenthesis.

^d^ Average maximum temperature increase over the baseline (37.8-38.6°C).

^e^ Relative inactivity index for the inoculated ferrets and for ferrets in the DC group.

^f^ Number of ferrets with nasal discharge, ocular discharge, or diarrhea over the total number of animals.

^g^ Number of animals euthanized during the experiment due to severe signs of illness over the total number of animals. Days of euthanasia are shown in parenthesis.

^h^ Number of contact ferrets with antibodies to homologous virus in serum (seroconversion) over the total number of ferrets from which sera was collected. *All ferrets with detectable A/Chile/25945/2023 A(H5N1) virus in NW did not survive the time course of infection; seroconversion was only tested in surviving animals.

NT-not tested.

### Systemic spread of HPAI A(H5N1) in ferrets

**Figure 3.**
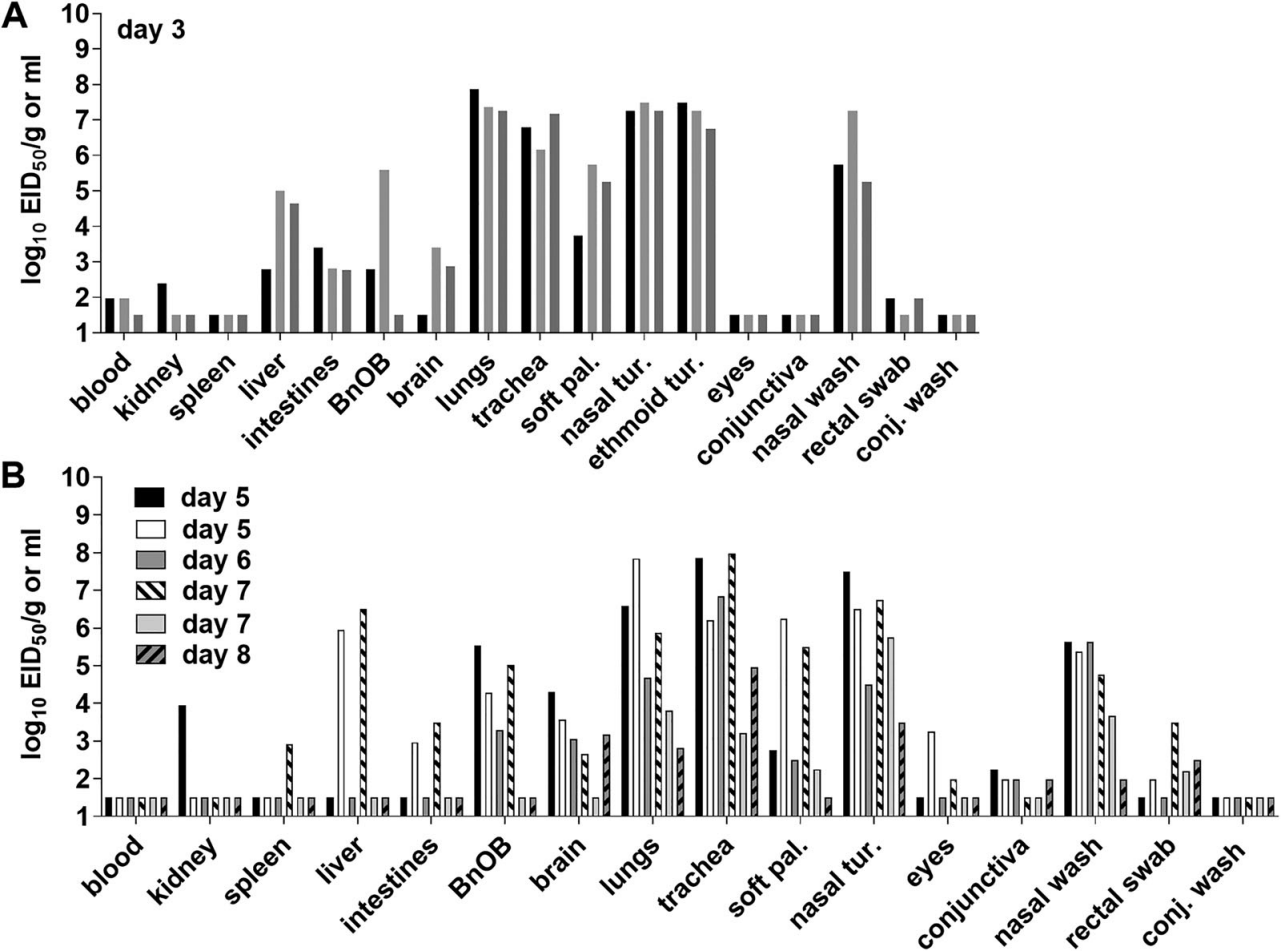
**Detection of A/Chile/25945/2023 A(H5N1) virus in ferret tissues**. Ferrets were inoculated with 6 log_10_ EID_50_ of virus; 3 ferrets were euthanized on day 3 post inoculation (A), and 6 ferrets were euthanized between days 5–8 due to severe clinical signs (B). Blood, soft palate (soft pal), nasal turbinate (nasal tur), ethmoid turbinate (ethmoid tur), eyes, conjunctiva, nasal wash, rectal swab, conjunctival wash (conj wash) viral titres are presented as log_10_ EID_50_/ml, and kidney, spleen, liver, intestines (pooled duodenum, jejuno-ileal loop, and descending colon), olfactory bulb (BnOB), brain (pooled anterior and posterior brain), lungs (each lobe sampled and pooled), and trachea are presented as log_10_ EID_50_/g of tissue. Bars represent individual ferrets. The limit of detection is 1.5 log_10_ EID_50_ per ml or g.

To assess systemic spread of the virus, three additional ferrets were inoculated with Chile/25945 A(H5N1) virus and euthanized on day 3 p.i. for tissue collection. The mean NW titre from these ferrets (6.8 log_10_ EID_50_/ml) was comparable to animals followed daily for clinical signs of infection. The virus replicated efficiently throughout the respiratory tract of all animals, with the highest titres detected in nasal turbinates, ethmoid turbinates, lungs, and trachea (6.1-8.5 log_10_ EID_50_/ml or gram). Virus was also present in 3/3 soft palate, intestine, and liver samples, 2/3 olfactory bulb, brain, and blood samples, and 1/3 kidney samples. Two out of three RS samples had virus at 1.98 log_10_ EID_50_/ml, while no virus was detected in ocular tissues or specimens collected on day 3 p.i. (Figure 3A).

To determine if ferrets sustained high titres in respiratory and extrapulmonary tissues beyond day 3 p.i., all ferrets that were euthanized between days 5–8 p.i. due to reaching humane endpoints, were subjected to standard necropsy. All animals had infectious virus detected throughout the respiratory tract (NW samples, nasal turbinates, trachea, and lung tissues). Five out of six ferrets had detectable virus in brain tissues, with sporadic detection in other extrapulmonary tissues (Figure 3B). These observations are consistent with other HPAI A(H5N1) viruses capable of replicating systemically in this model [16]. Collectively, these data support the capacity for Chile/25945 A(H5N1) virus to rapidly reach elevated viral titres throughout the respiratory tract and spread to extrapulmonary tissues, with titres sustained for the duration of the infection until euthanasia.

### Transmissibility of HPAI A(H5N1) human isolate in ferrets

**Figure 4.**
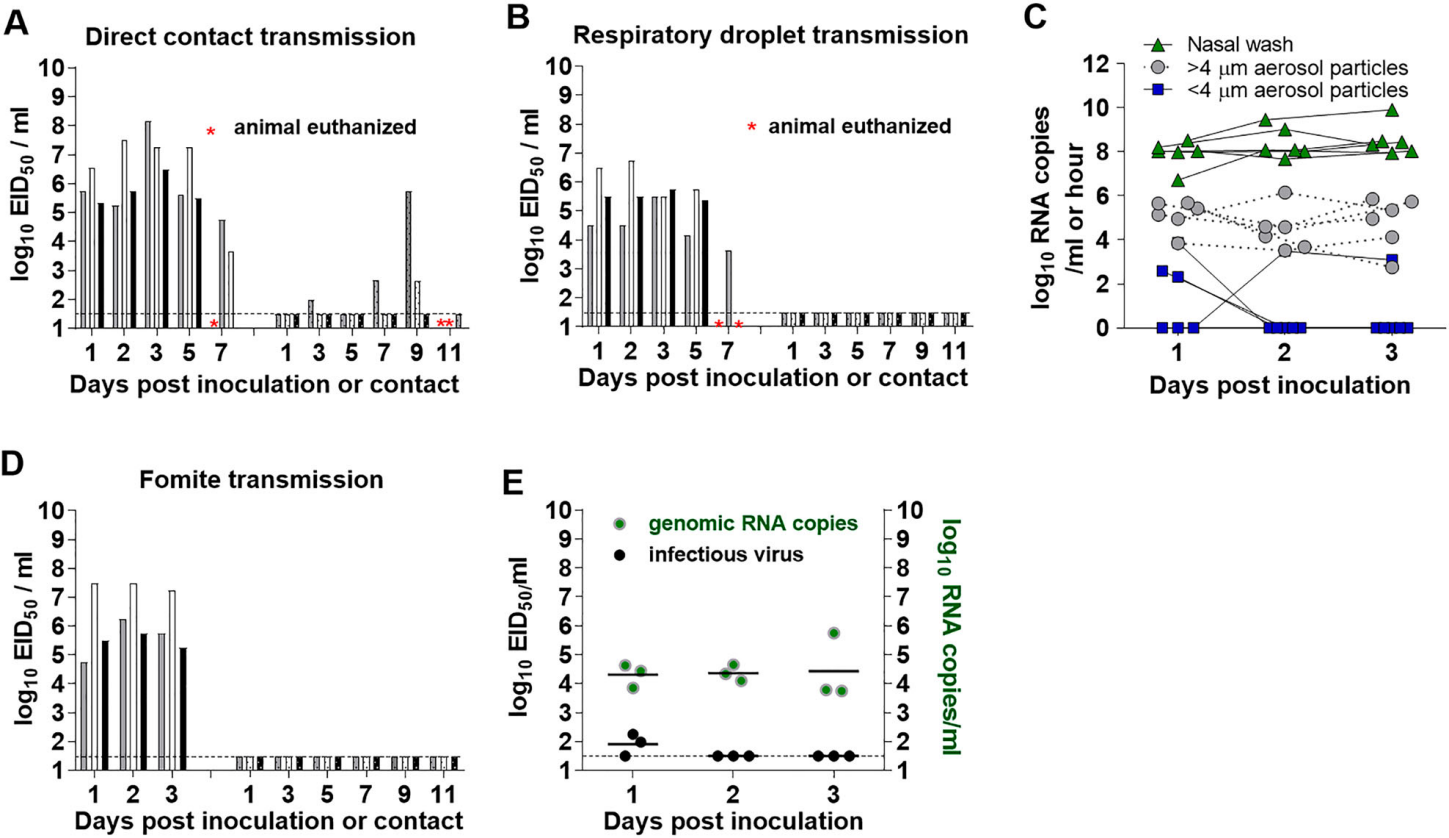
**Transmissibility of A/Chile/25945/2023 A(H5N1) virus in ferrets.** Six ferrets inoculated with 6.0 log_10_ EID_50_ of virus were used to test transmission in the presence of direct contact (A) or via respiratory droplets (B). Nasal wash samples were collected from both the inoculated (left side of each panel) and contact ferrets (right side of each panel) every other day post-inoculation or post-contact for titration in eggs. Bars represent individual ferrets. * Indicates the day when the animal was euthanized due to severe clinical signs of infection; viral titres in tissues for euthanized ferrets are shown in Figure 3B. Air samples were collected from each of the six inoculated ferrets for 1 h on days 1–3 post-inoculation and evaluated for the presence of viral RNA copies/hour of collection at 3.5 L/min. Grey circles represent viral RNA detected in particles of >4 µm, navy blue squares represent viral RNA detected in particles of <4 µm, green triangles represent viral RNA detected in nasal washes collected after aerosol sampling and are expressed as RNA copies/ml (C). Three additional inoculated ferrets were used to test for fomite transmission. The transmission experiment was conducted for 3 days, after which the inoculated ferrets were euthanized to test for virus tissue distribution (Figure 3A). Prior to swapping cages between inoculated and naïve ferret pairs, duplicate cage swabs were collected and subsequently assessed for the presence of infectious virus and viral RNA (E). The limit of detection was 1.5 log_10_ EID_50_ /ml (dashed line) and 210 RNA copies/hour of air collection at 3.5 L/min.

The ability of an avian virus to transmit between mammalian species represents an opportunity for the virus to adapt to humans and cause a pandemic. To address this concern, the capacity of Chile/25945 A(H5N1) virus to transmit to naïve ferrets was assessed in multiple settings. First, naïve ferrets were cohoused with virus-inoculated ferrets (at a 1:1 ratio) to ascertain the capacity for virus to transmit in the presence of direct contact. In this setting, 2/3 of the contact animals had infectious virus detected in NWs (Figure 4A). Both animals from which infectious virus was recovered developed severe disease symptoms, including fever, rapid weight loss, laboured breathing, nasal discharge, diarrhea, and lethargy, and were humanely euthanized on days 9 and 10 post-contact (p.c.) (Table 1). The third contact ferret did not have a detectable virus in NW specimens and did not display clinical signs of infection; however, seroconversion to homologous virus was detected 22 days p.c. (hemagglutination inhibition titre = 80), indicating that transmission occurred but did not result in a detectable disease outcome.

We next assessed the capacity of Chile/25945 A(H5N1) virus to transmit under more restrictive conditions. The capacity of this virus to transmit by the airborne route was tested by placing a naïve ferret in a cage adjacent to one housing an inoculated ferret, separated by perforated side walls, thus eliminating the potential for transmission by direct or indirect contact. In addition, to assess the extent to which virus-inoculated ferrets were releasing A(H5N1) virus into the air and the role this airborne release might contribute to onward transmission, we collected aerosol samples from 6 inoculated ferrets (3 donors in direct contact transmission and 3 donors in the airborne transmission model) once daily on days 1–3 p.i. and analyzed the load of viral RNA in the air. Consistent with a previous report [21], most of the viral RNA was collected in the >4 µm aerosol fraction, at a mean maximum of 5.7 ± 0.7 log_10_ RNA copies/hour of collection at 3.5 litres of air/min. Despite the presence of viral RNA detected from exhaled air of inoculated animals, no infectious virus was recovered from NW samples collected from contact animals, the animals did not present any clinical signs of infection, and no seroconversion to A(H5N1) virus was observed (Figure 4B).

Transmission via fomites was evaluated by exposing naïve contact animals to cages housing virus-inoculated animals without direct contact with inoculated animals. In other words, a naïve contact animal was placed into a cage that had previously held an inoculated ferret, so that the contact animal was exposed to stainless steel cage walls, bedding, food, and water that had been in direct contact with an inoculated ferret shedding virus into that environment via respiratory secretions, feces, and virus-laden aerosols (Figure 4D). Cage swaps were performed daily for the first three consecutive days between each of the three ferret pairs, such that contact animals were continuously housed in a cage that accommodated an inoculated ferret for the 24-hour period preceding the swap. Concurrently, prior to cage swap, cages housing inoculated ferrets were swabbed (one continuous swab across all 4 cage walls, floor, and water bottle sipper tube). Viral RNA from environmental swabs was detected at an average of 4.9 ± 0.6 log_10_ EID_50_/ml; infectious virus was only found in 2 cages on day 1 p.i. (1.98 log_10_ EID_50_/ml) (Figure 4E). Although some infectious virus was recovered from contaminated cage walls, NW specimens collected from contact animals did not contain detectable infectious virus. Furthermore, seroconversion was not observed at 22 days p.c., indicating that exposure of naïve contacts to contaminated cages for 3 days did not result in transmission of Chile/25945 A(H5N1) virus. Collectively, we found that the human case A(H5N1) virus was capable of transmission to contact animals, but only in the most permissive model encapsulating the widest range of transmission modes.

## Discussion

Extensive geographic expansion of clade 2.3.4.4b HPAI A(H5N1) viruses among wild birds and domestic poultry across Asia, Europe, Africa, North America, and South America in recent years has resulted in a great diversity of genotypically and phenotypically distinct viruses, including strains that have the ability to cross species barriers [22]. Mammalian infections with avian A(H5N1) are concerning as they create an opportunity for the virus to adapt to new hosts [6, 7]. While it appears that the clade 2.3.4.4b HPAI A(H5N1) viruses maintain an avian receptor binding preference [9], the presence of amino acid substitutions in the polymerase basic 2 protein (PB2) sequence of some mammalian isolates (available through GISAID) is suggestive of evolution towards more efficient replication in mammalian hosts, which often correlates with increased pathogenesis in mammalian models [23]. For example, viruses that caused outbreaks in mink in Spain possessed the PB2-T271A substitution, which enhances viral polymerase activity in mammalian cells, a substitution that has also been reported for 2009 swine-origin pandemic influenza viruses [3, 24]. When evaluated in mammalian models, two clade 2.3.4.4b recombinant mink viruses were shown to be highly virulent in mice and ferrets [25]. A severe disease outcome with neurological involvement was also reported in a study of 40 clade 2.3.4.4b A(H5N1) viruses, encompassing four different genotypes, isolated from free-living mesocarnivore species including red foxes, striped skunks, and mink in Canada. Some of these viruses also possessed mammalian adaptive mutations in PB2 (E627 K, E627 V and D701N) in addition to other known mammalian adaptation markers present in other proteins [26]. Similarly, PB2-E627K and D701N substitutions were also reported among viruses isolated from New England harbour and grey seals [4]. Viruses that caused outbreak in cats in Poland possessed two amino acid substitutions in the PB2 protein, Q526R and E627K; this combination of mutations was shown to mediate increased replication efficacy in mammalian cells and increased pathogenesis in mice [6, 7, 27]. The PB2 sequence of the human isolate studied here, Chile/25945 A(H5N1) virus, possessed Q591K and D701N substitutions, which were also present in some PB2 sequences obtained from outbreaks in sea lions in Peru and Chile in 2023 [5]. While we found that Chile/25945 virus exhibited more rapid growth in Calu-3 cells early after infection relative to other wild mammalian isolates examined, the ability for all of these viruses to reach high titres [exceeding those of a 2009 pandemic-derived A(H1N1)pdm09 virus] supports the high level of mammalian adaptation already present among zoonotic A(H5N1) viruses associated with avian-to-wild mammalian transmission, underscoring the need for continued investigation and evaluation of viruses with which humans may come into contact.

Sporadic human infections with clade 2.3.4.4b A(H5N1) viruses have been reported between 2021–2023 [9]; however, prior to this study, no evaluation has been done in mammalian models of any of the human isolates. The ferret is the most frequently used mammalian model for evaluating pathogenesis and transmissibility of newly emerging viruses [28]. Clade 2.3.4.4b A(H5N1) isolates have been shown to efficiently infect ferrets, causing illness ranging from mild to fatal depending on virus strain and genotype [16, 17, 25]. In this study, the Chile/25945 A(H5N1) virus displayed high pathogenicity in ferrets. The virus replicated efficiently in the ferret respiratory tissues, as evidenced by high virus titres in nasal turbinates, trachea, and lungs, resulting in nasal discharge and laboured breathing. Extensive extrapulmonary involvement was observed with some blood samples also testing positive for virus and further confirming systemic spread. The virus was detected in intestinal tissues and rectal swabs, and each of the inoculated ferrets experienced sustained diarrhea. Although obvious neurological signs were not observed within the short time course before humane euthanasia, the virus was also detected in brain tissues, indicating neurotropism. Two ferrets had heavy ocular discharge with one of the two ferrets having virus in a conjunctival wash specimen, in agreement with infectious virus recovered from eye and conjunctival tissue from virus-inoculated ferrets reaching humane endpoints. These results highlight the ability of Chile/25945 A(H5N1) viruses to efficiently replicate in pulmonary and extra pulmonary tissues of mammalian host leading to fatal disease. A future study is warranted to examine the role of known mammalian adaptation markers in the sequence of PB2 (such as Q591K and D701N) which may have contributed to the increased pathogenesis of this virus in ferrets, in addition to continued monitoring of clade 2.3.4.4b viruses for evolution and acquisition of novel mammalian adaptation markers.

Previously analyzed clade 2.3.4.4b A(H5N1) strains were not transmissible between ferrets [16, 25]. Here we show that the Chile/25945 A(H5N1) was able to transmit between co-housed ferrets. Two out of the three contacts tested positive for virus in their nasal washes and tissues, indicating productive transmission. The third contact had no detectable virus in any of the samples collected, but the sera showed evidence of exposure to the challenge virus as seroconversion was observed. When ferrets are co-housed, transmission can occur by direct contact between the infected and naïve ferret, by indirect contact via contaminated fomites (cage walls, bedding, etc.), or by inhalation of virus-laden particles. As supported by results from our fomite transmission and respiratory droplet transmission experiments, our data suggest that Chile/25945 A(H5N1) virus transmission likely occurred via close, direct ferret-to-ferret contact, rather than through contact with contaminated surfaces or inhalation. Despite the presence of Chile/25945 A(H5N1) viral RNA in the air, the transmission experiment results show that infectious load of virus in the air was not sufficient for the virus to transmit to ferrets housed in adjacent cages. Measurements of infectious virus in NW (mean maximum titres of 7.5 ± 1.1 log_10_ EID_50_/ml) as compared to RNA copy titres (mean maximum titres of 9.2 ± 0.7 log_10_ RNA copies/ml) suggest that levels of infectious virus in the air were at least 1.7 orders of magnitude lower than the detected RNA levels, though this remains to be experimentally confirmed. Despite the logistical challenges associated with this work, our findings support the need for additional efforts to quantify both infectious virus and viral RNA in air samples from inoculated ferrets [29]. Furthermore, studies such as this, which include assessments of multiple transmission modes, improve our understanding the factors contributing to onward transmission of virus.

Collectively, the clade 2.3.4.4b Chile/25945 A(H5N1) virus studied here displayed enhanced replicative ability in human respiratory tract cells and heightened transmissibility between ferrets in comparison to previously tested strains of the same clade isolated from avian and wild mammals [16, 25]. However, our results indicate the virus would require further adaptation to mammals to acquire an airborne transmissible phenotype and potentially become a pandemic virus. Nonetheless, this work warrants continuous monitoring of human adaptations in avian viruses, especially in strains isolated from humans, to aid pandemic preparedness efforts.

## Materials and methods

### Viruses

Stocks of HPAI A/Chile/25945/2023 A(H5N1) (Chile/25945) virus were propagated in the allantoic cavity of 10-day-old embryonated hens’ eggs at 37°C for 24-26 h. Allantoic fluid was pooled from multiple eggs, clarified by centrifugation, aliquoted and frozen at −80°C. To determine stock titre, samples were serially diluted and the 50% egg infectious dose (EID_50_) was calculated using the Reed and Muench method [30]. Each stock virus was sequenced and tested for exclusivity to rule out the presence of other subtypes of influenza virus. All research with HPAI viruses was conducted under biosafety level 3 containment, including enhancements required by the US Department of Agriculture and the Select Agent Program outlined in Biosafety in Microbiological and Biomedical Laboratories [31].

### Replication in Calu-3 cells

Calu-3 cells (ATCC, Manassas, VA, USA) were grown to confluence under air-liquid interface conditions in 12-mm–diameter transwell inserts (Corning, Corning, NY, USA). The cells were infected apically in triplicate at a multiplicity of infection of 0.01 for 1 hr, and then washed and incubated at 37°C or 33°C as previously described [32]. Timepoints were collected by adding 200 µl infection media to the apical side for 20 min prior to collection and subsequent storage at −80°C until titration. Virus titres in cell-supernatant samples were determined by a standard plaque assay in MDCK cells [33]. The limit of virus detection was 1 log_10_ PFU_50_/ml.

### Ferret Experiments

Animal research was conducted under the guidance of the Centers for Disease Control and Prevention's Institutional Animal Care and Use Committee in an Association for Assessment and Accreditation of Laboratory Animal Care International-accredited animal facility. Male Fitch ferrets (Triple F Farms, Sayre, PA) 6-months of age were used for this study. Each animal was confirmed serologically negative for currently circulating influenza A and B viruses by a standard hemagglutination inhibition assay. During experimentation, ferrets were housed in Duo-Flo Bioclean mobile units (Lab Products Incorporated, Seaford, DE). Nine ferrets were inoculated intranasally (1 ml) with 6 log_10_ EID_50_ of Chile/25945 A(H5N1) virus diluted in PBS. Twenty-four hours p.i. naïve ferrets were paired with inoculated ferrets to evaluate transmissibility of the virus via three transmission modes (three ferret pairs/transmission mode). In the direct contact transmission model, a naïve ferret was placed in the same cage as each inoculated ferret. In the respiratory droplet transmission model, a naïve ferret was placed in a cage adjacent to one housing an inoculated ferret allowing for air exchange between cages through perforated side cage walls (31). In the fomite transmission model, a naive ferret was exposed to a contaminated cage by swapping cages with an inoculated ferret once daily for three consecutive days. Prior to the swap, the cage housing an inoculated ferret was swabbed to test for the presence of virus on stainless-steel surfaces. Two cotton swabs were pre-moistened in PBS and the cage was swabbed in one continuous movement across all four stainless-steel cage walls, cage floor, and stainless-steel water bottle tube. The swabs were stored in PBS containing nasal wash solution and stored at −80°C until RNA extraction to evaluate viral RNA copy titres and titration in eggs for infectious virus titre evaluation. Inoculated and contact ferrets were observed daily for clinical signs of infection, and nasal washes, rectal swabs, and conjunctival washes were collected every 1–2 days for virus titre determination. Lethargy was measured based on a scoring system of 0–3 which was used to calculate a relative inactivity index as previously described [34, 35]. Three inoculated ferrets, which served as donors in the fomite transmission experiment, were euthanized on day 3 p.i. for the assessment of virus replication and systemic spread of the virus [36]. Animals that exhibited laboured breathing in addition to severe weight loss, lethargy, and diarrhea were humanely euthanized, and tissues were collected for titration.

### Aerosol collection

Air sampling was performed using a National Institute for Occupational Safety and Health (NIOSH) BC 251 two-stage cyclone aerosol sampler as previously described [37, 38]. Briefly, six alert ferrets (donors in the direct contact and respiratory droplet transmission experiments) were individually held in disinfected, vented transport containers (23.9 L in size) with a perforated lid for 1 h while air was collected at 3.5 L/min (210 L total collection volume) at the same time each day for 3 days. The samplers were attached to the outside of a container holding a single ferret with the sampler inlet protruding 3-4 cm into the container. The samplers collected three particle fractions: > 4 µm, 1–4 µm, and <1 µm aerodynamic diameter. Following collection, the samples were inactivated in AVL buffer (Qiagen) according to the manufacturer’s protocol. Samples were stored at −80°C before subsequent RNA extraction and quantification. Samplers were soaked in 70% ethanol, soaked, rinsed with distilled water, and air-dried after each sampling day. Transport containers were thoroughly decontaminated with 70% ethanol after each use.

### Statistical analysis

Experimental results were analyzed by two-way analysis of variance (ANOVA) followed by Tukey’s post-test. Analyses were performed using GraphPad Prism 7.0 software.

## Article summary line

An A(H5N1) influenza virus associated with human infection in Chile caused severe disease and displayed a capacity for close contact transmission in a mammalian model.

## References

[1] Lee DH, Bertran K, Kwon JH, et al. Evolution, global spread, and pathogenicity of highly pathogenic avian influenza H5Nx clade 2.3.4.4. J Vet Sci. 2017;18(S1):269–280. doi:10.4142/jvs.2017.18.S1.269 28859267 PMC5583414

[2] Gilbertson B, Subbarao K. Mammalian infections with highly pathogenic avian influenza viruses renew concerns of pandemic potential. J Exp Med. 2023;220(8). doi:10.1084/jem.20230447 PMC10276204 37326966

[3] Aguero M, Monne I, Sanchez A, et al. Highly pathogenic avian influenza A(H5N1) virus infection in farmed minks, Spain, October 2022. Euro Surveill. 2023;28(3):2300001. doi:10.2807/1560-7917.ES.2023.28.3.2300001 36695488 PMC9853945

[4] Puryear W, Sawatzki K, Hill N, et al. Highly pathogenic avian influenza A(H5N1) virus outbreak in New England seals, United States. Emerg Infect Dis. 2023;29(4):786–791. doi:10.3201/eid2904.221538 36958010 PMC10045683

[5] Leguia M, Garcia-Glaessner A, Munoz-Saavedra B, et al. Highly pathogenic avian influenza A (H5N1) in marine mammals and seabirds in Peru. Nat Commun. 2023;14(1):5489, doi:10.1038/s41467-023-41182-0 37679333 PMC10484921

[6] Domanska-Blicharz K, Swieton E, Swiatalska A, et al. Outbreak of highly pathogenic avian influenza A(H5N1) clade 2.3.4.4b virus in cats, Poland, June to July 2023. Euro Surveill. 2023;28(31). doi:10.2807/1560-7917.ES.2023.28.31.2300366 PMC10401911 37535474

[7] Rabalski L, Milewska A, Pohlmann A, et al. Emergence and potential transmission route of avian influenza A (H5N1) virus in domestic cats in Poland, June 2023. Euro Surveill. 2023;28(31). doi:10.2807/1560-7917.ES.2023.28.31.2300390 PMC10401914 37535471

[8] Briand FX, Souchaud F, Pierre I, et al. Highly pathogenic avian influenza A(H5N1) clade 2.3.4.4b virus in domestic Cat, France, 2022. Emerg Infect Dis. 2023;29(8):1696–1698. 37379514 10.3201/eid2908.230188 PMC10370847

[9] CDC. Technical Report: Highly Pathogenic Avian Influenza A(H5N1) Viruses 2023 [cited 2023 October 27]. Available from: https://www.cdc.gov/flu/avianflu/.

[10] Pulit-Penaloza JA, Sun X, Creager HM, et al. Pathogenesis and transmission of novel highly pathogenic avian influenza H5N2 and H5N8 viruses in ferrets and mice. J Virol. 2015;89(20):10286–93. doi:10.1128/JVI.01438-15 26223637 PMC4580194

[11] Kaplan BS, Russier M, Jeevan T, et al. Novel highly pathogenic avian A(H5N2) and A(H5N8) influenza viruses of clade 2.3.4.4 from North America have limited capacity for replication and transmission in mammals. mSphere. 2016;1(2):e00003-16. doi:10.1128/mSphere.00003-16 27303732 PMC4894690

[12] Richard M, Herfst S, van den Brand JM, et al. Low virulence and lack of airborne transmission of the Dutch highly pathogenic avian influenza virus H5N8 in ferrets. PLoS One. 2015;10(6):e0129827, doi:10.1371/journal.pone.0129827 26090682 PMC4474857

[13] Kim YI, Pascua PN, Kwon HI, et al. Pathobiological features of a novel, highly pathogenic avian influenza A(H5N8) virus. Emerg Microbes Infect. 2014;3(10):e75. 26038499 10.1038/emi.2014.75 PMC4217095

[14] Pulit-Penaloza JA, Brock N, Pappas C, et al. Characterization of highly pathogenic avian influenza H5Nx viruses in the ferret model. Sci Rep. 2020;10(1):12700, doi:10.1038/s41598-020-69535-5 32728042 PMC7391700

[15] Herfst S, Begeman L, Spronken MI, et al. A Dutch highly pathogenic H5N6 avian influenza virus showed remarkable tropism for extra-respiratory organs and caused severe disease but was not transmissible via air in the ferret model. mSphere. 2023;8(4):e0020023. 37428085 10.1128/msphere.00200-23 PMC10449504

[16] Kandeil A, Patton C, Jones JC, et al. Rapid evolution of A(H5N1) influenza viruses after intercontinental spread to North America. Nat Commun. 2023;14(1):3082, doi:10.1038/s41467-023-38415-7 37248261 PMC10227026

[17] Pulit-Penaloza JA, Belser JA, Brock N, et al. Pathogenesis and transmissibility of North American highly pathogenic avian influenza A(H5N1) virus in ferrets. Emerg Infect Dis. 2022;28(9):1913–1915. doi:10.3201/eid2809.220879 35840125 PMC9423912

[18] Castillo A, Fasce R, Parra B, et al. The first case of human infection with H5N1 avian Influenza A virus in Chile. J Travel Med. 2023;30(5). doi:10.1093/jtm/taad083 PMC10481412 37310882

[19] Pardo-Roa C, Nelson MI, Ariyama N, et al. Cross-species transmission and PB2 mammalian adaptations of highly pathogenic avian influenza A/H5N1 viruses in Chile. bioRxiv. 2023;55:739–768.

[20] Suttie A, Deng YM, Greenhill AR, et al. Inventory of molecular markers affecting biological characteristics of avian influenza A viruses. Virus Genes. 2019;55(6):739–768. doi:10.1007/s11262-019-01700-z 31428925 PMC6831541

[21] Pulit-Penaloza JA, Brock N, Belser JA, et al. Kinetics and magnitude of viral RNA shedding as indicators for Influenza A virus transmissibility in ferrets. Commun Biol. 2023;6(1):90, doi:10.1038/s42003-023-04459-0 36690690 PMC9871019

[22] Xie R, Edwards KM, Wille M, et al. The episodic resurgence of highly pathogenic avian influenza H5 virus. Nature. 2023;622(7984):810–817. doi:10.1038/s41586-023-06631-2 37853121

[23] Griffin EF, Tompkins SM. Fitness determinants of influenza A viruses. Viruses. 2023;15(9). doi:10.3390/v15091959 PMC10535923 37766365

[24] Bussey KA, Bousse TL, Desmet EA, et al. PB2 residue 271 plays a key role in enhanced polymerase activity of influenza A viruses in mammalian host cells. J Virol. 2010;84(9):4395–406. doi:10.1128/JVI.02642-09 20181719 PMC2863787

[25] Maemura T, Guan L, Gu C, et al. Characterization of highly pathogenic clade 2.3.4.4b H5N1 mink influenza viruses. EBioMedicine. 2023;97:104827, doi:10.1016/j.ebiom.2023.104827 37812908 PMC10579283

[26] Alkie TN, Cox S, Embury-Hyatt C, et al. Characterization of neurotropic HPAI H5N1 viruses with novel genome constellations and mammalian adaptive mutations in free-living mesocarnivores in Canada. Emerg Microbes Infect. 2023;12(1):2186608, doi:10.1080/22221751.2023.2186608 36880345 PMC10026807

[27] Song W, Wang P, Mok BW, et al. The K526R substitution in viral protein PB2 enhances the effects of E627K on influenza virus replication. Nat Commun. 2014;5:5509, doi:10.1038/ncomms6509 25409547 PMC4263149

[28] Belser JA, Katz JM, Tumpey TM. The ferret as a model organism to study influenza a virus infection. Dis Model Mech. 2011;4(5):575–9. doi:10.1242/dmm.007823 21810904 PMC3180220

[29] Belser JA, Pulit-Penaloza JA, Maines TR. Aerosolize this: Generation, collection, and analysis of aerosolized virus in laboratory settings. PLoS Pathog. 2023;19(3):e1011178, doi:10.1371/journal.ppat.1011178 36893118 PMC9997909

[30] Reed LJ, Muench H. A simple method of estimating fifty per cent endpoints. Am J Epidemiol 1938;27(3):493–497. doi:10.1093/oxfordjournals.aje.a118408

[31] Meechan PJ, Potts J. Centers for disease control and prevention. Biosafety in microbiological and biomedical laboratories 6th Edition 2020 [10/3/2023]. Available from: https://stacks.cdc.gov/view/cdc/97733.

[32] Zeng H, Goldsmith C, Thawatsupha P, et al. Highly pathogenic avian influenza H5N1 viruses elicit an attenuated type i interferon response in polarized human bronchial epithelial cells. J Virol. 2007;81(22):12439–49. doi:10.1128/JVI.01134-07 17855549 PMC2169033

[33] Szretter KJ, Balish AL, Katz JM. Influenza: propagation, quantification, and storage. Curr Protoc Microbiol. 2006;24(1–2):Chapter 15:Unit 15G 1. 10.1002/0471729256.mc15g01s3 18770580

[34] Reuman PD, Keely S, Schiff GM. Assessment of signs of influenza illness in the ferret model. J Virol Methods. 1989;24(1-2):27–34. doi:10.1016/0166-0934(89)90004-9 2760163

[35] Zitzow LA, Rowe T, Morken T, et al. Pathogenesis of avian influenza A (H5N1) viruses in ferrets. J Virol. 2002;76(9):4420–9. doi:10.1128/JVI.76.9.4420-4429.2002 11932409 PMC155091

[36] Maines TR, Lu XH, Erb SM, et al. Avian influenza (H5N1) viruses isolated from humans in Asia in 2004 exhibit increased virulence in mammals. J Virol. 2005;79(18):11788–800. doi:10.1128/JVI.79.18.11788-11800.2005 16140756 PMC1212624

[37] Pulit-Penaloza JA, Belser JA, Sun X, et al. Comparative assessment of severe acute respiratory syndrome coronavirus 2 variants in the ferret model. mBio. 2022;13(5):e0242122. 36135377 10.1128/mbio.02421-22 PMC9600705

[38] Blachere FM, Lindsley WG, Pearce TA, et al. Measurement of airborne influenza virus in a hospital emergency department. Clin Infect Dis. 2009;48(4):438–40. doi:10.1086/596478 19133798

